# Comorbidities of rheumatoid arthritis: Results from the Korean National Health and Nutrition Examination Survey

**DOI:** 10.1371/journal.pone.0176260

**Published:** 2017-04-19

**Authors:** Hyemin Jeong, Sun Young Baek, Seon Woo Kim, Yeong Hee Eun, In Young Kim, Hyungjin Kim, Jaejoon Lee, Eun-Mi Koh, Hoon-Suk Cha

**Affiliations:** 1 Department of Medicine, Samsung Medical Center, Sungkyunkwan University School of Medicine, Seoul, South Korea; 2 Biostatic and clinical epidemiology Center, Samsung Medical Center, Seoul, South Korea; Taipei Medical University, TAIWAN

## Abstract

This study aimed to evaluate the prevalence of comorbidities in patients with rheumatoid arthritis (RA) compared with the non-RA population. The 2010–2012 Korea National Health and Nutrition Examination Survey (KNHANES), which assesses the general health status of populations in South Korea using interviews and basic health assessment, was analyzed retrospectively. Weighted prevalence and odds ratio (OR) of comorbidities were analyzed in patients with RA compared with the non-RA population. The overall weighted (n = 37,453,158) prevalence of RA was 1.5%. Patients with RA were older and more female predominant than subjects without RA. The prevalence of living in an urban area, college graduation, alcohol consumption and smoking was lower in patients with RA than non-RA. Patients with RA had more comorbidities including hypertension, dyslipidemia, myocardial infarction (MI) or angina, stoke, osteoarthritis, lung cancer, colon cancer, pulmonary tuberculosis, asthma, diabetes, depression, thyroid disease and chronic kidney disease. After adjusting socioeconomic and lifestyle characteristics, RA was associated with an increased prevalence of MI or angina (OR 1.86, 95% CI 1.17–2.96, p = 0.009), pulmonary TB (OR 1.95, 95% CI 1.24–3.09, p = 0.004), asthma (OR 1.97, 95% CI 1.05–3.71, p = 0.036), thyroid disease (OR 1.71, 95% CI 1.05–2.77), depression (OR 2.38, 95% CI 1.47–3.85, p < 0.001) and hepatitis B (OR 2.34, 95% CI 1.15–4.80, p = 0.020) compared with the non-RA population. Prevalence of solid cancer was not significantly associated with RA after adjustment.

## Introduction

Rheumatoid arthritis (RA) is a chronic inflammatory disease and with dominant features of joint inflammation and damage. RA is associated with progressive disability, systemic complications, and socioeconomic costs [1]. Moreover, survival of patients with RA is worse than survival of the general population [2]. Reah et al. reported that RA patients often die of cardiovascular disease in the long-term observational study [3]. The higher death rate of patients with RA appears to be the consequence of more serious co-morbid conditions [4]. RA is associated with an increased prevalence of several comorbidities [5]. Comorbidities frequently seen in patients with RA include cardiovascular disease, infection, malignancy, lung disease and neuropsychiatric disease [6–9]. Long-term systemic inflammation in RA may promote comorbidity or medications used to treat RA may be associated with comorbidity. In addition, other traditional risk factors such as smoking could cause comorbidity [10].

The increased prevalence of comorbidities, as well as RA itself, is associated with an economic burden on patients, their families, and society [11]. The elderly RA patients who also have emerging comorbidities present a unique challenge to treating clinicians [12]. Although occurrence of comorbidities is more common in RA than controls, comorbidity is often underrecognized and undertreated [13,14]. Detecting and managing specific comorbidities and preventing their development are important and recommended by the European League Against Rheumatism (EULAR) [15].

Several studies addressing comorbidities in RA have been published; however, some studies lack a comparator group without RA and other studies were performed adjusting only for age and gender. Moreover, the data regarding medical comorbidities associated with RA in previous studies were mainly from western populations. The aim of this study was to determine the prevalence of comorbidities in Korean adult population with RA compared with the non-RA population, with adjusting for socioeconomic and lifestyle characteristics using data from the 2010–2012 Korea National Health and Nutrition Examination Survey (KNHANES).

## Method

### Study population and data collection

The KNHANES is a nation-wide survey that has been conducted periodically by the Korea Centers for Disease Control and Prevention to investigate the health and nutritional status of the Korean population. It assesses the general health and nutrition status of populations in South Korea using interviews about health and nutrition, and a basic health assessment. Participants were selected using proportional allocation-systemic sampling with multistage stratification to derive a representative Korean population. Although individual participants were not equally representative of the Korean population, this survey provides representative estimates of the noninstitutionalized Korean civilian population by using the power of sample weight. Every year, 10,000 to 12,000 individuals in about 3,800 households are selected from a panel based on the National Census Data. The participation rate of selected households in the past several cycles of KNHANES has been high, ranging from 79% to 84%. Written informed consent was obtained from all participants before completing the survey. This study was approved by the interstitial review board of Samsung Medical Center.

The KNHANES was conducted by four special research teams, each composed of eight experts including nurses, nutritionists, and persons who major in public health. The selected professional investigator was placed at the investigation site after completing one month of education and practice. Subsequently, the ability to conduct research was verified through regular education and on-site quality management. A standardized interview was performed with an established questionnaire in the homes of the participants to collect information on demographic variables. The established questionnaire consisted of the demographic and socioeconomic characteristics of the subjects. Data on age, gender, income, region, education, marital status, alcohol consumption, and smoking status were collected. Alcohol consumption was divided into 4 groups based on the frequency of alcohol consumption during the last one year; never, 1 ≤ week, 2–3 /week, and 4 ≥ week. Income level was divided by quartile for average of individual monthly income. Urban and rural areas were classified by administrative district. RA was defined in the questionnaire as “RA diagnosed by a physician” through a standardized interview. The question was, “Was your RA diagnosed by a physician?” The questionnaire consists of three responses (1. Yes, 2. No 3. I have never been sick before). Participants who choose 1 (Yes) were classified as RA. The interview was conducted individually by a trained professional investigator. Classification criteria for RA were not applied in defining RA in this study. Medical comorbidities were defined in the same way as the RA diagnosis. Information was collected on cardiovascular comorbidities including hypertension, dyslipidemia, diabetes, stroke, myocardial infarction or angina that were diagnosed by a physician. Information was also collected on malignancies including stomach cancer, colon cancer, breast cancer, cervical cancer, and lung cancer and other medical comorbidities including osteoarthritis, pulmonary tuberculosis, thyroid disease, depression, atopic dermatitis, depression, chronic kidney disease, hepatitis B, hepatitis C, and liver cirrhosis that were diagnosed by a physician. Classification criteria for osteoarthritis was not applied in defining osteoarthritis. Definition of osteoarthritis and other medical comorbidities were dependent on the information provided by the participants in the interview. Height and weight were assessed using standardized techniques and equipment. Height was measured to the nearest 0.1 cm using a portable stadiometer (Seriter, Bismarck, ND, USA). Weight was measured to the nearest 0.1 kg using a Giant-150N calibrated balance-beam scale (Hana, Seoul, Korea). Body mass index (BMI) was calculated by dividing weight by the square of height (kg/m^2^).

### Statistical analyses

To reflect representative estimates of the noninstitutionalized Korean civilian population, the survey sample weights, which were calculated by taking into account the sampling rate, response rate, and age/sex proportions of the reference population (2005 Korean National Census registry), were applied in all of the analyses. Univariable logistic regression analysis was performed to compare clinical characteristics between subjects with RA and subjects without RA. Multivariable logistic regression models were computed with comorbidities as a dependent variable, and RA as an independent variable, adjusting for socioeconomic and lifestyle characteristics (age, sex, income, region, marital status, drinking, smoking, BMI). Odds ratios (ORs) with 95% confidence intervals (CIs) of RA for each of the comorbidities were calculated. P-values were corrected by Bonferroni’s method for multiple testing. Statistical analyses were performed using SAS (SAS version 9.4; SAS Institute, Cary, NC, USA). All *P*-values were two-sided, and *P* < 0.05 was considered statistically significant.

## Results

Among 25,534 participants in the 2010–2012 KNHANES, 19,599 participants who were 19 years of age or older were selected. Then, 1,712 participants who had missing data for diagnosis of RA were excluded. In total, 17,887 participants were selected for analysis. The frequency of missing data for each item was very low, with a maximum of about 1%. The weighted (n = 37,453,158) demographics and clinical characteristics of study population are presented in Table 1. The mean age of participants was 45.3 years, and the percentage of female was 50.8%. The prevalence of college graduates was 32.2% and the mean BMI was 23.7 kg/m^2^. The prevalence of RA was 1.5%.

**Table 1:** Baseline characteristics of the study population (unweighted, n = 17,887; weighted, n = 37,453,158).

	Weighted % or Mean ± SD	Missing data, unweighted n (%)
Age, years	45.3 ± 0.2	0 (0.00)
Women	50.8	0 (0.00)
Income		195 (1.09)
Low	26.9	
Mid-low	25.6	
Mid-high	24.7	
High	22.9	
Region		0 (0.00)
Urban	79.3	
Rural	20.7	
Education		29 (0.16)
Elementary school	18.8	
Middle school	10.1	
High school	38.9	
College graduation	32.2	
Marital status		6 (0.03)
Married	77.8	
Unmarried	22.2	
Alcohol consumption		129 (0.72)
Never	23.1	
1 ≤ week	53.4	
2–3 /week	16.0	
4 ≥ week	7.5	
Smoking		55 (0.31)
Never smoker	53.7	
Ex-smoker	19.8	
Current smoker	26.5	
Body mass index (kg/m^2^)	23.7 ± 0.0	92 (0.51)
Rheumatoid arthritis	1.5	0 (0.00)
Hypertension	17.0	0 (0.00)
Dyslipidemia	8.2	0 (0.00)
Diabetes	6.3	0 (0.00)
Stroke	1.3	0 (0.00)
Myocardial infarction or angina	1.9	0 (0.00)
Myocardial infarction	0.7	0 (0.00)
Lung cancer	0.1	4 (0.02)
Cervical cancer	0.3	4 (0.02)
Breast cancer	0.4	4 (0.02)
Colon cancer	0.3	4 (0.02)
Stomach cancer	0.5	4 (0.02)
Osteoarthritis	8.1	0 (0.00)
Pulmonary tuberculosis	4.2	0 (0.00)
Asthma	3.0	0 (0.00)
Thyroid disease	3.1	4 (0.02)
Atopic dermatitis	3.0	3 (0.02)
Depression	3.8	3 (0.02)
Chronic kidney disease	0.3	3 (0.02)
Hepatitis B	1.5	3 (0.02)
Hepatitis C	0.2	3 (0.02)
Liver cirrhosis	0.3	4 (0.02)

Clinical characteristics were analyzed according to the presence of RA (Table 2). Subjects with RA were older and more female predominant compared with subjects without RA. Subjects with RA lived more in the rural areas and had a lower educational level. The weighted prevalence of current smoker was lower and never smoker was higher in subjects with RA compared with non-RA subjects. In addition, alcohol consumption was less common in subjects with RA than in the non-RA population. The prevalence of cardiovascular risk factors or cardiovascular disease, including hypertension, dyslipidemia, diabetes, stroke, myocardial infarction or angina, myocardial infarction, and angina was increased in patients with RA compared with the non-RA group. Among solid malignancies, colon cancer and lung cancer were more common in the RA group. In addition, other medical comorbidities, including osteoarthritis, pulmonary tuberculosis, asthma, diabetes, thyroid disease, depression, and chronic kidney disease were more common in subjects with RA compared with subjects without RA.

**Table 2:** Weighted clinical characteristics based on the presence of rheumatoid arthritis in the Korean adult population.

	Non-RA (Weighted n = 36,874,636)	RA (Weighted n = 578,522)	OR (95% CI)	P value
Age, years, mean ± SD	45.1 ± 0.2	57.5 ± 1.0	1.05 (1.04–1.06)	< 0.001
Female, %	50.4	76.9	3.27 (2.28–4.69)	< 0.001
Income, %				0.516
Low	26.9	27.6	Reference	
Mid-low	25.6	23.8	0.9 (0.57–1.42)	1.000 (adj.)
Mid-high	24.6	28.2	1.11 (0.71–1.72)	1.000 (adj.)
High	22.9	20.4	0.87 (0.55–1.37)	1.000 (adj.)
Region, %				
Urban	80.3	74.2	Reference	
Rura	19.7	25.8	1.43 (1.10–1.86)	0.008
Education, %				< 0.001
Elementary school	18.5	43.8	Reference	
Middle school	10.0	16.9	0.71 (0.44–1.17)	0.300 (adj.)
High school	39.1	24.5	0.26 (0.18–0.39)	< 0.001 (adj.)
College graduation	32.4	14.8	0.19 (0.12–0.32)	< 0.001 (adj.)
Marital status, %				
Married	77.5	95.7	Reference	
Unmarried	22.5	4.3	0.16 (0.07–0.38)	< 0.001
Alcohol consumption, %				< 0.001
Never	22.8	41.0	Reference	
1 ≤ week	53.6	42.5	0.44 (0.31–0.63)	< 0.001 (adj.)
2–3 /week	16.0	13.4	0.46 (0.25–0.86)	0.009 (adj.)
4 ≥ week	7.6	3.1	0.48 (0.01–0.56)	< 0.001 (adj.)
Smoking, %				
Never smoker	53.4	71.7	Reference	< 0.001
Ex-smoker	19.80	18.5	0.70 (0.46–1.06)	0.102
Current smoker	26.8	9.8	0.27 (0.15–0.48)	< 0.001
Body mass index (kg/m^2^), mean ± SD	23.7 ± 0.0	23.9 ± 0.2	1.02 (0.99–1.05)	0.294
Hypertension, %	16.8	30.3	2.15 (1.62–2.86)	< 0.001
Dyslipidemia, %	8.1	14.1	1.86 (1.34–2.57)	< 0.001
Diabetes, %	6.21	12.9	2.24 (1.54–3.25)	< 0.001
Stroke, %	1.3	2.8	2.25 (1.16–4.38)	0.017
Myocardial infarction or angina, %	1.9	5.7	3.19 (2.06–4.94)	< 0.001
Myocardial infarction, %	0.6	1.4	2.26 (0.10–5.13)	0.051
Angina, %	1.3	4.5	3.58 (2.18–5.87)	< 0.001
Lung cancer, %	0.1	0.5	8.84 (1.15–67.99)	0.036
Cervical cancer, %	0.3	0.8	2.91 (0.95–8.89)	0.061
Breast cancer, %	0.4	0.7	2.13 (0.64–7.04)	0.216
Colon cancer, %	0.3	1.0	3.48 (1.23–9.89)	0.019
Stomach cancer, %	0.5	0.5	1.05 (0.22–5.00)	0.952
Osteoarthritis, %	7.9	22.6	3.42 (2.51–4.66)	< 0.001
Pulmonary tuberculosis, %	4.2	8.6	2.15 (1.36–3.40)	0.001
Asthma, %	3.0	7.3	2.57 (1.44–4.59)	0.002
Thyroid disease, %	3.0	8.0	2.79 (1.76–4.43)	< 0.001
Atopic dermatitis, %	3.0	1.5	0.48 (0.21–1.11)	0.087
Depression, %	3.7	11.2	3.27 (2.09–5.13)	< 0.001
Chronic kidney disease, %	0.3	1.1	3.43 (1.30–9.02)	0.013
Hepatitis B, %	1.5	2.9	1.98 (0.99–3.95)	0.052
Hepatitis C, %	0.2	0.4	2.26 (0.30–16.99)	0.427
Liver cirrhosis, %	0.3	0.5	1.75 (0.24–12.94)	0.583

P-values were adjusted by Bonferroni’s method for multiple testing.

On adjusting socioeconomic and lifestyle characteristics (age, sex, income, region, education, marriage, alcohol drinking, smoking, BMI), RA was associated with an increased prevalence of myocardial infarction or angina (OR 1.86, 95% CI 1.17–2.96, p = 0.009). Angina was significantly related to RA rather than myocardial infarction. Prevalence of solid cancer was not significantly associated with RA after adjustment. Among other medical comorbidities, RA was associated with an increased prevalence of pulmonary tuberculosis (OR 1.95, 95% CI 1.24 to 3.09, p = 0.004), asthma (OR 1.97, 95% CI 1.05–3.71, p = 0.036), thyroid disease (OR 1.71, 95% CI 1.05–2.77), depression (OR 2.38, 95% CI 1.47–3.85, p < 0.001) and hepatitis B (OR 2.34, 95% CI 1.15–4.80, p = 0.020) compared with the non-RA population (Table 3).

**Table 3:** Adjusted ORs (95% CI) for medical comorbidities among patients with RA compared with non-RA.

	OR (95% CI)	P value
Hypertension	0.97 (0.67–1.36)	0.873
Dyslipidemia	1.11 (0.78–1.57)	0.581
Diabetes	1.33 (0.87–2.02)	0.189
Stroke	1.27 (0.65–2.47)	0.490
Myocardial infarction or angina	1.86 (1.17–2.96)	0.009
Myocardial infarction	1.67 (0.73–3.83)	0.228
Angina	1.88 (1.11–3.17)	0.018
Lung cancer	7.61 (0.92–63.28)	0.060
Cervical cancer	1.29 (0.43–3.84)	0.648
Breast cancer	0.95 (0.28–3.20)	0.928
Colon cancer	2.06 (0.72–5.90)	0.179
Stomach cancer	0.72 (0.14–3.67)	0.689
Osteoarthritis	1.28 (0.90–1.81)	0.173
Pulmonary tuberculosis	1.95 (1.24–3.09)	0.004
Asthma	1.97 (1.05–3.71)	0.036
Thyroid disease	1.71 (1.05–2.77)	0.030
Depression	2.38 (1.47–3.85)	< 0.001
Atopic dermatitis	0.99 (0.41–2.40)	0.987
Chronic kidney disease	1.96 (0.73–5.25)	0.180
Hepatitis B	2.34 (1.15–4.80)	0.020
Hepatitis C	1.47 (0.18–11.80)	0.714
Liver cirrhosis	1.22 (0.16–9.38)	0.846

Adjusting for age, sex, income, region, education, marriage, drink, smoking, body mass index

## Discussion

We evaluated the comorbidities of patients with RA using a nationwide survey conducted by the Korean government. The most frequently associated comorbidities in patients with RA included hypertension (30.3%), osteoarthritis (22.6%), dyslipidemia (14.1%), diabetes (12.9%), depression (11.2%), pulmonary tuberculosis (8.6%), thyroid disease (8.0%), asthma (7.3%), and myocardial infarction or angina (5.7%). These data partly correspond with COMORA, an earlier international, cross-sectional study, which reported that depression (15%), asthma (6.6%), and cardiovascular events (6%) were the most frequently associated diseases with RA [6].

The results of our study indicated that Korean RA patients showed characteristics of a healthier lifestyle than the general population. The percentage of ex-smokers in the RA and non-RA groups was similar. However, the percentage of current smokers was significantly lower and never-smoking was significantly higher in subjects with RA, as compared with the non-RA population. Previous studies reported that the prevalence of cigarette smoking was higher in RA compared with the non-RA population; in addition, RA patients had a higher prevalence of cardiovascular disease than non-RA patients [10,16–19]. However, several studies showed that smoking was not significantly different between RA and non-RA subjects, although cardiovascular disease was increased in the RA group [20–22]. Roman et al. reported that carotid atherosclerosis was significantly increased in the RA group, despite a more favorable traditional risk factor profile in the RA group than control; moreover, the percentage of current smoker was significantly lower in the RA group than control [23]. In the nationwide RA registry across South Korea (KORean Observational study Network for Arthritis, KORONA), non-smokers comprised 84.2% (3962/4707) and current smokers 8.0% (377/4707) of the RA patients [24]. Non-smoking rates of KORONA is high, which is similar to those of the current KNHANES. Considering that most of the studies were performed in western countries, significantly lower smoking rates in RA was characteristic of Korean patients. Comparative characteristics of Korean patients with RA with an international, cross-sectional study (COMORA) indicated that the proportion of current smoker and patients with Framingham score > 20% was significantly lower in the Korean RA population compared with international data (p < 0.001) [25]. In addition, the prevalence of metabolic syndrome was higher in patients with RA in the United States and Europe [26]. In contrast, the prevalence of metabolic syndrome was lower in Korean RA patients compared with healthy control after adjustment of lifestyle characteristics [27]. Smoking is a known environmental risk factor for RA [28]. Smoking reportedly causes an immune response to citrullinated proteins in humans with HLA-DRB1 shared epitope (SE), a risk gene for RA, and contributes to anti-cyclic citrullinated peptide (anti-CCP) antibody production [29]. A study on the western population indicated that smoking exposure increases the risk factor for anti-CCP antibody in SE positive patients with RA, but not in SE negative patients [30]. However, Korean studies have shown that smoking increased RA susceptibility in individuals with SE alleles, regardless of their ACPA or RF status [31]. This discrepancy may be caused by ethnic difference. The most significant HLA-DRB1 allele for RA susceptibility is *0401 in Caucasians [32,33]. However, HLA-DRB1 *0405 allele is the most significant for RA in Koreans and Asian populations [34–36]. In particular, HLA-DRB1 *0401 allele confers the highest risk for occurrence of anti-CCP antibodies [37]. In an animal model, citrullinated peptide binds to the *0401 allele in transgenic mice with a high affinity [38]. Thus, the effect of smoking on the RA susceptibility may be different in Korean and Caucasian populations due to genetic difference of SE alleles. Although HLA-DRB1 has been associated with the RA population, many other risk loci have been shown to confer RA susceptibility. There is ethnic difference in other genetic loci. For example, although protein tyrosine phosphatase-22 (*PTPN22)* gene has been associated with RA in Caucasians, it does not play a role in Korean and Asian populations with RA [39–41]. In contrast, peptidylarginine deiminase 4 (*PADI4*), solute carrier family 22 member 4 (*SLC22A4)*, and Fc receptor like 3 (*FCRL3)* genes have been well documented among Asian populations, but not among the western Europeans [42–46]. Considering the higher non-smoking rate in Korean RA patients, environmental factors other than smoking or genetic backgrounds may be associated with the pathogenesis of RA in Korean patients. Further study is needed to clarify the different pathogenetic mechanisms in RA between the Korean and western populations.

In the current study, alcohol consumption was lower in subjects with RA. This is consistent with the recent meta-analysis that reported that low-to-moderate alcohol consumption was inversely associated with the development of RA, suggesting a protective effect [47,48]. Alcohol has been suggested to have protective effects in the development of other chronic diseases [49]. However, a population-based cohort study in Sweden showed that there was no significant association of alcohol consumption and the risk of RA development [50]. The association between RA and alcohol consumption remains unclear.

It is well known that cardiovascular disease is increased among RA patients, as compared with the general population [7,51]. The increased risk of cardiovascular disease in patients with RA is comparable to the risk in patients with type-2 diabetes mellitus [52,53]. Cardiovascular disease is the leading cause of death among RA patients, who show a 50% higher risk than the general population [54,55]. In the current study, most of cardiovascular disease and risk factors were significantly increased in patients with RA. Myocardial infarction or angina was significantly related to RA after adjustment of socioeconomic and lifestyle characteristics, corroborating the results from the UK population-based nationwide study [56]. Definitions of RA and cardiovascular comorbidities were based on self-report forms, and the OR of cardiovascular disease was 1.52. Thus, the results of the current study were similar to the other major western dataset [57].

In the current study, patients with RA had an increased prevalence of lung cancer and colon cancer, as compared with the non-RA population. However, there was no significant relationship between cancer and RA after adjustment. A recent meta-analysis study showed that patients with RA were at an increased risk of lung cancer and lymphoma compared with the general population [58]. Colorectal and breast cancers showed a decrease in risk among patients with RA compared with the general population; whereas, cervical cancer, prostate cancer, and melanoma appeared to have no consistent trend in risk [58]. Chronic lung inflammation due to disease in patients with RA may be associated with the increased risk of lung cancer. It is well known that smoking increases the risk of both RA and lung cancer. In contrast to western countries, squamous cell cancer is the most frequent cell type in Korea, followed by adenocarcinoma. Lung cancer occurs predominantly in male smokers [59]. Different characteristics of lung cancer between races might explain the lack of an increased risk of lung cancer in Korean RA patients.

Among other medical comorbidities, RA was significantly associated with pulmonary tuberculosis, asthma, thyroid disease, depression and hepatitis B. The prevalence of pulmonary tuberculosis in this study was 4.2%. WHO reports that the incidence rates of active tuberculosis in South Korea in 2015 are 80 per 100,000 population [60]. Considering incidence rates of active tuberculosis prevalence in the WHO data, not only active tuberculosis patients but also latent or treated tuberculosis patients may have been included in the KNHANES. There was no information on extra-pulmonary Tb in the KNHANES. Prevalence of pulmonary tuberculosis is significantly increased in patients with RA. These data correspond well with the previous study results indicating that RA is associated with an increased risk of tuberculosis [61]. In the current study the adjusted OR was 2.0, which is similar to those of the population-based nationwide studies conducted in Taiwan (OR 2.0) and in Sweden (OR 2.0) [62,63]. A compromised immune system could greatly increase an individual’s susceptibility to tuberculosis infection or an underlying immune disturbance could predispose to the development of RA as well as tuberculosis. In addition, the recently increased use of biologic agents in RA treatment may have attributed to the elevated risk [62,64].

In the current study, the prevalence of asthma was significantly increased in patients with RA. Several studies reported that the prevalence of asthma was lower in RA patients than in control groups [65–67]. However, these studies showed no statistical significance and the sample size was small. In contrast, the cumulative incidence of asthma was significantly higher in children with RA compared with children without RA [68]. Shen et al. reported that patients with RA had a significantly higher risk of developing asthma than control patients in all sex and age subgroups in a population-based cohort study [68]. The balance between Th1 and Th2 cells is critical in inflammatory and immune disorders. RA is classified as a Th1 immune disease; whereas, asthma involves the activation of Th2 cells [69,70]. Hartung et al reported that a Th2-mediated atopic disease protects against the development of Th1-mediated RA [71]. Our study results do not support the concept of Th1/Th2 mutual antagonism. The pathogenesis of RA and asthma is complex, involving genetic as well as other environmental factors. The cause of increased incidence of asthma in RA is uncertain. However, these data indicated that the Th1 and Th2 diseases can coexist, suggesting that a common environmental denominator might involve the disease processes.

We found that RA was associated with an increased risk of thyroid disease. In the KANHNES survey, the type of thyroid disease was not investigated. The most common thyroid dysfunction in RA patients is of an autoimmune nature accompanied by elevated thyroid autoantibody titers. In addition, the concurrent affection of joint and thyroid gland is probably related to a genetic predisposition, most often HLA-DR [72]. Chan et al. suggested that thyroid function and thyroid peroxidase antibody (TPO Ab) tests should be performed in RA patients; and thyroid function follow-up is needed especially in high risk groups (female, raised TSH, positive TPO Ab) [73].

RA is a chronic illness that has physical as well as psychological effects. Comorbid depression is common in patients with RA, with a prevalence of 15%–42% [6,74,75]. The 11% prevalence rate for depression in the current study was slightly lower than previously reported. Low socioeconomic status, female gender, younger age, functional limitation, pain, and systemic inflammation status have all been linked to depression among patients with RA. Patients with RA and depression have worse health outcomes, including poor medication adherence, increased health service utilization, pain, disability and death [76]. In the current study, the adjusted OR of depression was the highest. Thus, the understanding of socioeconomic factors and individual patient characteristics could facilitate more comprehensive treatment in patients with RA.

In the current study, RA was associated with an increased prevalence of hepatitis B. This corresponds well with the recent nationwide data in Taiwan, which reported that RA patients had an increased risk of hepatitis B infection after adjustment for potential risk factors [77]. Extrahepatic manifestation of hepatitis B infection includes serum sickness-like syndrome, vasculitis, skin rash, arthritis, and glomerulonephritis [78]. The pathogenesis of HBV-associated arthritis is attributed to the deposition of immune complexes containing viral antigens (HBV surface antigen HBsAg or HBeAg) and their respective antibodies (anti-HBs and anti-HBe) in synovial tissues [79]. Reactivation of viral hepatitis due to the use of immunosuppressive drugs might also contribute to the increased risk of hepatitis in RA patients.

The present study has some limitations. First, the different diagnoses were dependent on the information provided by the participants in the interview. Patients may misunderstand the information given by physicians. In this study, classification criteria were not applied for the definition of RA. This is a major limitation of this type of nationwide survey. The prevalence of RA in this study was 1.5%. Considering that the prevalence of RA is approximately 1% of the worldwide population [80,81], the prevalence of RA in the current study might be overestimated. The definition of osteoarthritis and other medical comorbidities was also dependent on the questionnaires without objective confirmatory data. Although trained researchers asked the study population on the diagnosis during the face-to-face interviews, the risk of recall bias should be considered. Therefore, causality could not be inferred due to the cross-sectional design of the study and the limitations about definition of RA and other comorbidities. Second, because our study used data from a nationwide survey, we could not assess the disease activity or medications for RA and were unable to evaluate the association between the disease activity of RA and comorbidities. We were unable to analyze the association between biologic agent and prevalence of infection such as tuberculosis and hepatitis. Third, we could not analyze the prevalence of lymphoma because “diagnosis of lymphoma” was not included in the investigating items of KNHANES. And the confidence interval of lung cancer is wide due to a small number of subjects with lung cancer in total, especially in RA group. In addition, we analyzed the prevalence of RA and other medical comorbidities. Prevalent cohort is often used rather than incident cohort in disease of chronic nature. Studying a prevalent exposure, rather than an incident one, has been shown to result in a bias for some causal effects that change with time. This might affect the results and should be considered when interpreting the outcomes of this study. The strength of our study is that we analyzed large, nationally representative data of the Korean adult population. In addition, we analyzed the comorbidities of RA adjusting both socioeconomic and lifestyle characteristics. Despite the limitations of our study, the current study provides a comprehensive cross-sectional data of RA and comorbidities for Asian populations.

In conclusion, the overall prevalence of RA was 1.5% and patients with RA had healthier lifestyle characteristics than the non-RA population in Korea. Our study showed that RA was associated with an increased risk of cardiovascular disease, pulmonary tuberculosis, asthma, thyroid disease, hepatitis B, and depression after adjustment of socioeconomic and lifestyle characteristics. In addition, the prevalence of solid cancer was not significantly associated with RA. These findings collectively suggest that individuals with RA might be at risk of several medical comorbidities; therefore, clinicians need to pay attention to emerging comorbidities in patients with RA. Further study is required to verify the prevalence of medical comorbidities in patients with RA based on specific classification criteria of RA. We believe that the present study will provide important information for further research on the association of comorbidities and RA.
