# Correction: Comorbidities of rheumatoid arthritis: Results from the Korean National Health and Nutrition Examination Survey

**DOI:** 10.1371/journal.pone.0178309

**Published:** 2017-05-18

**Authors:** Hyemin Jeong, Sun Young Baek, Seon Woo Kim, Yeong Hee Eun, In Young Kim, Hyungjin Kim, Jaejoon Lee, Eun-Mi Koh, Hoon-Suk Cha

There is an error in Table 2. The “P value” for the row “Smoking, %” is incorrectly presented in the “Never smoker” row. Please see the corrected Table 2 here.

**Table 2:** Weighted clinical characteristics based on the presence of rheumatoid arthritis in the Korean adult population.

	Non-RA (Weighted n = 36,874,636)	RA (Weighted n = 578,522)	OR (95% CI)	P value
Age, years, mean ± SD	45.1 ± 0.2	57.5 ± 1.0	1.05 (1.04–1.06)	< 0.001
Female, %	50.4	76.9	3.27 (2.28–4.69)	< 0.001
Income, %				0.516
Low	26.9	27.6	Reference	
Mid-low	25.6	23.8	0.9 (0.57–1.42)	1.000 (adj.)
Mid-high	24.6	28.2	1.11 (0.71–1.72)	1.000 (adj.)
High	22.9	20.4	0.87 (0.55–1.37)	1.000 (adj.)
Region, %				
Urban	80.3	74.2	Reference	
Rura	19.7	25.8	1.43 (1.10–1.86)	0.008
Education, %				< 0.001
Elementary school	18.5	43.8	Reference	
Middle school	10.0	16.9	0.71 (0.44–1.17)	0.300 (adj.)
High school	39.1	24.5	0.26 (0.18–0.39)	< 0.001 (adj.)
College graduation	32.4	14.8	0.19 (0.12–0.32)	< 0.001 (adj.)
Marital status, %				
Married	77.5	95.7	Reference	
Unmarried	22.5	4.3	0.16 (0.07–0.38)	< 0.001
Alcohol consumption, %				< 0.001
Never	22.8	41.0	Reference	
1 ≤ week	53.6	42.5	0.44 (0.31–0.63)	< 0.001 (adj.)
2–3 /week	16.0	13.4	0.46 (0.25–0.86)	0.009 (adj.)
4 ≥ week	7.6	3.1	0.48 (0.01–0.56)	< 0.001 (adj.)
Smoking, %				< 0.001
Never smoker	53.4	71.7	Reference	
Ex-smoker	19.80	18.5	0.70 (0.46–1.06)	0.102 (adj.)
Current smoker	26.8	9.8	0.27 (0.15–0.48)	< 0.001 (adj.)
Body mass index (kg/m^2^), mean ± SD	23.7 ± 0.0	23.9 ± 0.2	1.02 (0.99–1.05)	0.294
Hypertension, %	16.8	30.3	2.15 (1.62–2.86)	< 0.001
Dyslipidemia, %	8.1	14.1	1.86 (1.34–2.57)	< 0.001
Diabetes, %	6.21	12.9	2.24 (1.54–3.25)	< 0.001
Stroke, %	1.3	2.8	2.25 (1.16–4.38)	0.017
Myocardial infarction or angina, %	1.9	5.7	3.19 (2.06–4.94)	< 0.001
Myocardial infarction, %	0.6	1.4	2.26 (0.10–5.13)	0.051
Angina, %	1.3	4.5	3.58 (2.18–5.87)	< 0.001
Lung cancer, %	0.1	0.5	8.84 (1.15–67.99)	0.036
Cervical cancer, %	0.3	0.8	2.91 (0.95–8.89)	0.061
Breast cancer, %	0.4	0.7	2.13 (0.64–7.04)	0.216
Colon cancer, %	0.3	1.0	3.48 (1.23–9.89)	0.019
Stomach cancer, %	0.5	0.5	1.05 (0.22–5.00)	0.952
Osteoarthritis, %	7.9	22.6	3.42 (2.51–4.66)	< 0.001
Pulmonary tuberculosis, %	4.2	8.6	2.15 (1.36–3.40)	0.001
Asthma, %	3.0	7.3	2.57 (1.44–4.59)	0.002
Thyroid disease, %	3.0	8.0	2.79 (1.76–4.43)	< 0.001
Atopic dermatitis, %	3.0	1.5	0.48 (0.21–1.11)	0.087
Depression, %	3.7	11.2	3.27 (2.09–5.13)	< 0.001
Chronic kidney disease, %	0.3	1.1	3.43 (1.30–9.02)	0.013
Hepatitis B, %	1.5	2.9	1.98 (0.99–3.95)	0.052
Hepatitis C, %	0.2	0.4	2.26 (0.30–16.99)	0.427
Liver cirrhosis, %	0.3	0.5	1.75 (0.24–12.94)	0.583

P-values were adjusted by Bonferroni’s method for multiple testing.
